# Infantile Cytomegalovirus-Associated Severe Warm Autoimmune Hemolytic Anemia: A Case Report

**DOI:** 10.3390/children4110094

**Published:** 2017-11-03

**Authors:** Hassan K. Khalifeh, Youmna M. Mourad, Cynthia T. Chamoun

**Affiliations:** 1 Department of Pediatric Hematology Oncology, Zahraa University Hospital, Beirut 1131, Lebanon; drhasskha@yahoo.com (H.K.K.); youmna_mourad@hotmail.com (Y.M.M.); 2 Department of Pediatric Hematology Oncology, Faculty of Medical Sciences, Lebanese University, Beirut 1131, Lebanon

**Keywords:** autoimmune hemolytic anemia, direct antiglobulin testing, cytomegalovirus

## Abstract

Autoimmune hemolytic anemia is a rare hematologic entity in children. Etiologies are mainly viruses or bacteria. We describe here a case of severe warm autoimmune hemolytic anemia (IgG- and C3d-positive direct antiglobulin test) in an immunocompetent 6-month-old infant with acute Cytomegalovirus infection that responded well to corticotherapy and intraveneous immunoglobulins without using blood component transfusion. This case demonstrates the importance of recognizing CMV in infantile Autoimmune Hemolytic Anemia, especially because hemolysis can be severe and lethal.

## 1. Introduction

Auto-immune hemolytic anemia (AIHA) is characterized by the presence of autoantibodies that bind to the erythrocyte surface membrane and lead to premature red cell destruction. AIHA is a rare condition in infants and young children with an annual incidence of 0.2 per 100,000 in subjects less than 20 years old [1]. However, it is still the main cause of acquired extra-corpuscular hemolysis in children [2]. According to pathophysiologic behavior of the autoantibodies, we classify the AIHA into warm (WAIHA) with ideal autoantibody susceptibility at 37 °C, cold (CAIHA) with ideal autoantibody susceptibility at 4 °C and paroxysmal cold hemoglobinuria (PCH) [3]. The WAIHA constitutes 70–90% of all infantile AIHA and is caused mainly by IgG antibodies [1,2]. It can be divided into primary or secondary AIHA. The latter is due to lymphoproliferative and autoimmune disorders, drugs and solid malignancies) [3]. In children, it is often acute, with 80% good prognosis merely by using short-term steroid therapy. In children younger than two years of age or in teenagers, there might be either resistance to steroids or dependence on high-dose steroids [4]. Viruses and bacteria can be associated with AIHA in children [5]. Cytomegalovirus (CMV) is a common viral agent responsible for a wide range of manifestations. However, the mechanisms responsible for the correspondent immune response remains unknown [6]. CMV’s clinical manifestations are widely variable and depend on whether the patient is immunosuppressed or not and they are related to a direct viral cytotoxic effect on specific organs (e.g., gastrointestinal tract, retina, and hematopoietic system). Hematologically, it may manifest as transient neutropenia and thrombocytopenia or it may appear more severe, such as in AIHA. Immunocompromised patients are the victims of severe hemolytic manifestations of the disease, albeit rare in itself [5,7]. There are reports of some immunocompetent adults who have hemolytic disease caused by CMV infection [6]. We describe here a severe hemolytic anemia in an immunocompetent 6-month-old child with acute CMV infection that responded to corticotherapy and IVIG without any blood component transfusion.

## 2. Case

A 6-month-old male infant, previously healthy, was admitted for jaundice, tea colored urine and decreased oral intake with a history of ten days of afebrile upper respiratory tract infection for which he received clarithromycin for atypical organisms with dexamethasone Per Os syrup for symptomatic relief of cough. He has a negative history for familial hematologic diseases.

In the physical exam, there was marked pallor, icteric sclera with no hepatosplenomegaly and no pathological lymphadenopathies. He had normal blood pressure with a heart rate of 150 (mildly tachycardic for his age) and no tachypnea. Investigations revealed, on complete blood count (CBC), severe anemia with hemoglobin 4.5 g/dL (11–13 g/dL), MCV 120 fl (70–86 fl), platelets 80,000/mm^3^ (150,000–450,000/mm^3^) and high reticulocyte percentage (65%)(normal value < 1%). White count and differential were normal. The peripheral blood smear showed anisopoikilocytosis, macrocytosis, no hypochromia, polychromatophilia, numerous nucleated red blood cells, few spherocytes were present and schistocytes were absent. His biochemical studies showed increased indirect bilirubin (1.18 mg/dL) (normal value < 1mg/dL), decreased haptoglobin (0.02 g/L) (0.2–0.03 g/L), increased LDH (1323 U/L) (normal value < 280U/L), normal creatinine (0.23 mg/dL) (normal value < 1 mg/dL), normal folate and vitamin B12 levels. The immunohematological studies showed positive direct antiglobulin testing (DAT) using polyspecific antiglobulins (anti-IgG and anti-C3d) and positive DAT using the specific anti-IgG monoclonal antiglobulin. The indirect antiglobulin testing (IAT) was also positive and all cross matched blood units were incompatible. The titer of the autoantibody was very high (>1/40,960) with a strength of +4 and did not decrease until four months after the event. Immunoglobulins (IgG, IgM, and IgA) quantitation was normal. Antinuclear antibodies profile and rheumatic factor were also negative. Viral serology (Epstein-Barr virus, human immunodeficiency virus, hepatitis B and C viruses, adenoviruses.) was all normal except for CMV, which was positive—IgM titer was very high (nine times the upper limit) and the IgG titer was slightly above the upper limit. CMV PCR was also positive. The patient was managed medically without any packed red cell transfusion because he was relatively stable and constantly monitored. An intraveneous bolus of 4 mg/kg of methylprednisolone was given on day 0. A dose of 0.5 g/kg per day (half the required dose for financial reasons) of intraveneous immunoglobulins (IVIG) was given on day 1 plus a dose of 2 mg/kg per day of methylprednisolone that was continued alone for the next seven days before shifting to oral prednisone (2 mg/kg per day) for three weeks. Then, a progressive prednisone tapering over the next five months was planned. No specific CMV-immunoglobulins or antivirals were available. The hemoglobin level increased to 5.4 g/dL the second day, then progressively until 8.5 g/dL before discharge (time course of five days) and before shifting to oral prednisone. We lost sight of the patient as his parent did not bring him back for follow up until a month after stopping prednisone. He had a hemoglobin level of 11 g/dL and a platelet count of 230,000/mm^3^ at the time.

## 3. Discussion

Here, we report a case of a 6-month-old male infant with severe AIHA (Hb = 4.5 g/dL) associated with primary CMV infection. AIHA secondary to antibiotic intake (clarithromycin) was ruled out since the CMV titers were so elevated. Multiple clinicopathological parameters must be considered in the diagnosis of this disease including the presence of anemia, autoagglutination, spherocytes, positive IAT and the elimination of any other underlying causes of anemia. No single finding is pathognomonic for AIHA, so careful interpretation is important. AIHA in childhood may be associated with malignant and autoimmune diseases as well as syndromes of immunodeficiency [1,8].

CMV serology may not be obtained regularly in a patient with hemolysis, so the true incidence of this disorder is underestimated [6]. Unlike that in adults, where acute post infectious forms are of the isolated C3d type of DAT, in children, CMV infection can be associated with warm antibody AIHA. We consider our case to be warm AIHA because DAT is positive for both C3d and IgG [3]. Mixed AIHA (C3d and IgG) represents 32% of AIHA in children [2]. They usually evolve chronically with severe hemolytic crises. The IgG/IgG + C3d DAT was significantly associated with a lower rate of continuous complete remission. The C3d isolated AIHA was associated with a good prognosis, even with a shorter follow-up, since the two-year continuous complete remission rate was 71% [2]. It is important to note that intense positivity of DAT in our patient (titer > 1/40,960) correlated with the intensity of the hemolysis. However, it is not always the case [2].

For the very anemic child with warm-reactive AIHA, intraveneous methylprednisolone should be administered every 6 h at a dose of 1–2 mg/kg for the first 24–72 h. Oral prednisone at a total dose of 1–2 mg/kg per day is then used after the child is more clinically stable. Typically, high doses are used for two to four weeks, followed by a slow taper over two to six months, based upon the hemoglobin concentration, reticulocyte count, and direct antiglobulin test results. Using this approach has resulted in an overall response rate of approximately 80% [9].

Corticosteroids work by inhibiting Fc receptor mediated clearance of erythrocytes that carry IgG in the spleen, and by inhibiting synthesis of autoantibodies [10]. Some children exhibit resistance to steroids or dependence on high dose steroids, especially if they are younger than two years of age or teenagers. They would also develop typical steroid severe side effects on growth, bone development, and the endocrine system. These primary AIHA children have a mortality rate of 10% [11,12].

IVIG is used in pediatric patients with AIHA who only have a partial response to steroids. IVIG works by intensively blocking the reticuloendothelial system; they thus inhibit phagocytosis of red cells carrying IgG [8]. Anti-CD20 monoclonal antibody has been used in some children with either primary or secondary AIHA that are refractory to or dependent on corticosteroids with success [13,14,15].

Splenectomy is an alternative therapeutic option for patients with AIHA who require high maintenance prednisolone doses or who have multiple and frequent relapses [2]. Immunomodulatory drugs such as Danazol, Azathioprine, Alemtuzumab, cyclosporine, mycophenolate mofetil and chemotherapeutic agents like high dose cyclophosphamide and vincristine have been used for refractory cases as well [16,17,18].

In this case, there has been a good response even after the first dose of corticosteroids and there has been a rise in hemoglobin level despite the severe hemolytic crisis without the need for transfusion. In fact, unstable patients with severe anemia should be transfused slowly and cautiously with the least incompatible blood available, monitoring closely for signs of increased hemolysis, as the presence of autoantibodies can mask alloantibodies. Stable patients with severe anemia should be monitored closely and transfused if they show signs of hemodynamic instability or worsening anemia. The extent of hemolysis and response to treatment will be monitored as time passes by the use of those laboratory tests initially employed for establishing the diagnosis of hemolysis (e.g., LDH, haptoglobin, indirect bilirubin, DAT test) during the five months of tapering of oral Prednisone [19,20]. The prognosis is expected to be excellent despite the clinical and laboratory findings.

## 4. Conclusions

This case emphasizes that, in children, autoantibody titers can be remarkably elevated for a lengthy period of time and still be associated with a good prognosis.
